# Cardioprotective Mechanisms of Beta-Blockers in Myocardial Ischemia and Reperfusion: From Molecular Targets to Clinical Implications

**DOI:** 10.3390/ijms26209843

**Published:** 2025-10-10

**Authors:** Athina Nasoufidou, Marios G. Bantidos, Barbara Fyntanidou, Christos Kofos, Panagiotis Stachteas, Alexandra Arvanitaki, Paschalis Karakasis, Marios Sagris, George Kassimis, Nikolaos Fragakis, Efstratios Karagiannidis

**Affiliations:** 1 Second Department of Cardiology, Aristotle University of Thessaloniki, Hippokration General Hospital of Thessaloniki, 54642 Thessaloniki, Greece; 2 Department of Emergency Medicine, Aristotle University of Thessaloniki, AHEPA General University Hospital of Thessaloniki, 54636 Thessaloniki, Greece; 3 Department of Cardiology, Tzaneio General Hospital of Piraeus, 18536 Piraeus, Greece

**Keywords:** apoptosis, beta-blockers, cardioprotection, endothelial function, inflammation, ischemia, ischemic heart disease, oxidative stress, reperfusion, translational research

## Abstract

Ischemic heart disease remains the leading cause of death despite substantial advances in diagnosis, revascularization therapies, and risk-factor control. Beta-adrenergic receptor blockers (Beta-Blockers, BBs), long used to control heart rate, blood pressure, and reduce arrhythmic risk, may also confer cardioprotection through mechanisms beyond hemodynamic unloading. This review integrates an extensive range of preclinical, translational, and clinical studies to present a comprehensive overview of the cardioprotective effects of BBs in the context of myocardial ischemia and reperfusion injury. Mechanistic domains include modulation of redox homeostasis, attenuation of inflammation and neutrophil activation, preservation of mitochondrial integrity and anti-apoptotic signaling, improvement of endothelial function, and stabilization of calcium handling. Third-generation compounds, carvedilol and nebivolol, demonstrate additional antioxidant and vasodilatory benefits compared with first- and second-generation agents; however, no consistent class-wide effect exists across most pathways. The evidence base remains fragmented, often derived from agent- or context-specific studies in heterogeneous populations, with uncertainty surrounding optimal timing of intervention. By bridging mechanistic understanding with clinical outcomes, this review highlights the importance of standardized assessment of BB effects, the development of personalized treatment approaches, and the pursuit of future research to address ongoing translational gaps.

## 1. Introduction

Ischemic Heart Disease (IHD) remains the leading cause of death and disability, despite decades of advancement in diagnostic, interventional, and pharmacological strategies [1,2]. While its clinical manifestations are well characterized, key pathophysiological aspects are yet to be elucidated; myocardial ischemia extends far beyond overt obstructive Coronary Artery Disease (CAD). Ischemia/Reperfusion (I/R) triggers a complex cascade involving cellular stress, inflammatory signaling, and metabolic disruption [3]. Consequently, persisting residual morbidity, recurrent events, and progression to Heart Failure (HF) highlight therapeutic gaps insufficiently addressed and motivate adjunctive, mechanism-based cardioprotection.

Beta-adrenergic receptor blockers (Beta-Blockers, BBs) have played a foundational role in the treatment of IHD since their introduction in the 1960s, initially prescribed to alleviate symptoms of Angina Pectoris (AP) and control arrhythmias [4]. BBs have been associated with a substantial reduction in all-cause mortality and Major Adverse Cardiovascular Events (MACE) in patients with HF with reduced Ejection Fraction (EF, HFrEF) following Acute Myocardial Infarction (AMI), benefits that have cemented their status in cardiovascular pharmacotherapy [5,6]. These outcomes have largely been attributed to the blockade of β-adrenergic receptors, resulting in decreased Heart Rate (HR), Blood Pressure (BP), myocardial oxygen demand, and mitigation of sympathetic overdrive.

Over time, it has become increasingly evident that the benefits of BBs may extend beyond hemodynamic unloading. An expanding body of experimental and translational research suggests that these agents influence deeper biological processes involved in ischemic injury and repair, including the regulation of inflammatory pathways, oxidative stress, mitochondrial function, and cell survival [7]. However, such pleiotropic effects are not uniformly present across all compounds; differences in receptor selectivity, lipophilicity, ancillary pharmacology, and even the extent of preclinical and clinical investigation contribute to significant heterogeneity [8,9]. Therefore, a consistent class-wide effect cannot be assumed for most pathways [10]. Among the various β-blockers, carvedilol and nebivolol demonstrate the most robust antioxidant, anti-inflammatory, and endothelial-modulating properties, whereas atenolol and propranolol exhibit weaker or inconsistent effects [11]. Recognizing these agent-specific properties is important, as they may influence clinical outcomes beyond traditional hemodynamic effects and should be considered when selecting a β-blocker for targeted cardioprotection.

In light of this complexity, there is a pressing need to move beyond fragmented, drug-specific studies and toward a comprehensive evaluation and categorization of BB cardioprotection. This review aims to integrate mechanistic, preclinical, and clinical perspectives to support the evolving bench-to-bedside trajectory and offer a more coherent framework for guiding future research and therapeutic BB strategies against ischemia-related injury.

## 2. Current Classification and Main Actions

BBs are structurally and pharmacodynamically heterogeneous, with classification traditionally based on receptor selectivity and ancillary properties. Three generations are recognized: non-selective (first-generation), β1-selective (second-generation), and agents with vasodilatory or additional pleiotropic effects (third-generation) [11].

### 2.1. First-Generation Beta-Blockers

First-generation BBs, exemplified by propranolol, are non-selective antagonists of β1- and β2-adrenergic receptors. Their clinical effects are primarily mediated through blockade of the β1–G stimulatory (Gs)–adenylyl cyclase–cyclic Adenosine Monophosphate (cAMP)–Protein Kinase A (PKA) signaling axis, primarily in cardiomyocytes (reducing contractility, prolonging relaxation, and decreasing lusitropy) and juxtaglomerular cells (suppressing renin release) [12,13]. However, concurrent β2 antagonism [which included both G inhibitory (Gi) and Gs pathways] can impair vasodilatory and bronchodilatory signaling. This may lead to unopposed increases in peripheral vascular resistance, heightened risk of bronchospasm, and diminished counter-regulatory responses to hypoglycemia in insulin-treated patients [14,15,16]. In addition, propranolol’s high lipophilicity allows Central Nervous System (CNS) penetration, which may contribute to fatigue, sleep disturbance, and mood changes [17].

### 2.2. Second-Generation Beta-Blockers

Second-generation BBs (e.g., atenolol, metoprolol) exhibit greater β1 selectivity, often referred to as “cardioselectivity.” This property, while dose-dependent, has a marked therapeutic advance over non-selective agents [18,19]. Atenolol is hydrophilic, with limited CNS penetration and primary renal elimination, whereas metoprolol is more lipophilic and undergoes hepatic metabolism [11]. Both effectively lower HR and BP and are commonly prescribed in hypertension, CAD, and post-MI settings [20]. However, unlike third-generation agents, they do not provide vasodilatory and/or antioxidant benefits, limiting their mechanistic reach and weakening their impact on hard clinical outcomes [11]. This lack of pleiotropic properties may explain why these older agents have shown weaker or inconsistent results in long-term clinical trials compared to third-generation agents [21].

### 2.3. Third-Generation Beta-Blockers

The development of third-generation BBs reflects the need for benefits beyond β1 selectivity (e.g., improved endothelial function) [22,23]. They are defined by the addition of pleiotropic properties to standard β1 blockade, extending from α1-adrenergic antagonism, β3-adrenergic activation, and enhancement of endothelial Nitric Oxide (NO) signaling to targeting fibrotic, inflammatory, and redox-sensitive pathways [17,21]. Unlike earlier generations, these agents can improve vascular tone independently of HR or contractility [24,25]. Carvedilol and nebivolol are the most widely studied representatives, and their distinct properties are discussed in detail in Section 4.

## 3. Ischemia-Induced Myocardial Remodeling

### 3.1. Initial Ischemic Changes at the Cellular Level

Ischemia typically begins when a coronary artery becomes partially or completely blocked, triggering structural and cellular changes, leading to overall cardiac remodeling. Reduced blood flow depletes Adenosine Triphosphate (ATP), disrupts ion balance, and impairs cell viability; if prolonged, this leads to mitochondrial and sarcolemmal rupture, calcium imbalance, and membrane damage, which trigger inflammation. While reperfusion is vital for cell survival, it can also cause injury by generating ROS and aggravating calcium overload [26]. Necrosis—recognized by the presence of contraction bands and inflammation—accounts for the majority of cell death after an MI. However, multiple pathomechanisms have been identified to contribute to myocardial damage, like apoptosis or autophagy [27].

### 3.2. Adverse Myocardial Remodeling

Massive cell death triggers an immune response with inflammatory cells disintegrating the extracellular matrix and expanding the infarct area, leading to thinning and dilation of the ventricle. Consequently, fibroblasts will replace the damaged tissue with collagen, and neurohormonal activation via the Renin–Angiotensin–Aldosterone System (RAAS) and Sympathetic Nervous System (SNS) will further promote fibrosis and cell death, resulting in cardiac remodeling and increased HF risk [28]. Adverse remodeling occurs in two phases: the early phase involves cell dilation, necrosis, and fibrosis localized to the infarcted region, while the late phase affects viable myocardium surrounding the infarct, where increased wall stress triggers dilatation and hypertrophy [29].

According to Laplace’s law, Left Ventricular (LV) wall stress is directly proportional to the pressure within the ventricle and its radius and inversely proportional to the myocardial wall thickness [30]. In the early phase of MI, the infarcted region stretches due to unopposed forces from the contracting healthy myocardium, causing wall thinning and infarct expansion. This dilation leads to volume and pressure overload in the remaining healthy myocardium. During the late phase, to compensate for increased workload, viable cardiomyocytes hypertrophy and lengthen to maintain stroke volume. However, excessive overstretching disrupts the Frank–Starling mechanism, exacerbating LV dilation. This process creates a self-perpetuating cycle of increasing wall stress, dilation, and wall thinning [31].

### 3.3. Biochemical Mechanisms of Adverse Remodeling

#### 3.3.1. Cellular Changes

In the myocardium under normal oxygen conditions, energy is primarily derived from free fatty acids and carbohydrates through β-oxidation and glycolysis. The resulting metabolites enter the mitochondria, where oxidative phosphorylation couples with the Krebs cycle to generate ATP, the main energy source of the cardiomyocyte [32].

During ischemia, oxygen availability is reduced, preventing mitochondria from producing sufficient ATP. As a compensatory response, the heart shifts to anaerobic glycolysis, producing lactate with much lower energy yield. Prolonged ischemia causes lactate accumulation, intracellular acidosis, and inhibition of glycolytic enzymes, further impairing ATP generation [33]. The resulting energy depletion disrupts ionic homeostasis, promotes calcium overload, and drives cardiomyocyte death [34]. Sustained ischemia causes irreversible injury, but timely reperfusion restores oxygen, allowing aerobic metabolism and ATP production to recover in stunned but viable myocardium, with gradual restoration of contractile function [35].

Despite its benefits, reperfusion may worsen injury, as the sudden oxygen supply triggers excess Reactive Oxygen species (ROS) [36], which damage cellular structures—especially the sarcoplasmic reticulum—disrupt calcium handling, and activate inflammatory pathways that recruit immune cells and cytokines, extending tissue injury [37].

Ischemia–reperfusion is a double-edged sword: timely reperfusion salvages myocardium but also triggers oxidative and inflammatory stress that promote remodeling and dysfunction [37].

#### 3.3.2. Extracellular Matrix Changes

The cardiac Extracellular Matrix (ECM) provides structural support and plays a central role in remodeling after ischemia. After MI, fibroblasts infiltrate the infarct to replace necrotic tissue and stabilize the wall, but excessive ECM deposition increases stiffness, reduces compliance, and raises arrhythmic risk [38,39]. In the infarcted myocardium, collagen types I and III forming the dominant scar components. Reperfusion further stimulates expression of proteins such as intermediate Cartilage Layer Protein 1 (CILP1), asporin, Adipocyte Enhancer-Binding Protein 1 (AEBP1), and Transforming Growth Factor β-Induced (TGFBI) gene-h3 [40]. Ultimately, both reparative fibrosis (scar formation) and reactive fibrosis (diffuse interstitial remodeling) can compromise ventricular function [33].

#### 3.3.3. Inflammation

Dying cardiomyocytes release proinflammatory and chemoattractant cytokines that recruit neutrophils, macrophages, and lymphocytes to the injured myocardium. Neutrophils degrade collagen via metalloproteinases, macrophages clear necrotic debris and secrete cytokines that stimulate fibroblast activation, while lymphocytes help sustain the inflammatory response for weeks [40]. Toll-Like Receptors (TLR2 and TLR4) also mediate this immune activation, and their inhibition has been shown in animal models to reduce fibrosis and improve outcomes. Within hours, interleukins like IL-6, IL-1β, and Tumor Necrosis Factor-α (TNF-α) are upregulated, promoting apoptosis, ECM remodeling, and structural changes [41]. While inflammation is essential for repair, excessive or prolonged activation exacerbates adverse remodeling and promotes fibrosis [42].

#### 3.3.4. Endothelin

Ischemia and inflammatory cytokines induce Endothelin-1 (ET-1), which promotes adverse remodeling by activating macrophages, stimulating cytokine release upregulating adhesion molecules, and driving neutrophil aggregation. ET-1 also directly induces cardiomyocyte hypertrophy, further worsening remodeling [29].

#### 3.3.5. Neurohormonal Regulation

Rapid SNS activation, triggered by ischemia, assists in maintaining adequate cardiac output by increasing heart rate and contractility. β-adrenergic receptor stimulation elevates cAMP and activates protein kinase B (PKB), enhancing Nitric Oxide (NO) signaling and providing short-term cardioprotection [43]. However, sustained SNS overactivity leads to LV hypertrophy, impaired excitation–contraction coupling, apoptosis, fibrosis, oxidative stress, and further RAAS activation.

Through Angiotensin II type 1 (AT1) receptors, angiotensin II drives collagen deposition, apoptosis, and hypertrophy, while Angiotensin II type 2 (AT2) receptor signaling appears protective [44]. Thus, sustained SNS and RAAS activity drive maladaptive remodeling and are central pharmacologic targets [45].

## 4. Molecular and Cellular Mechanisms of Expanded Beta-Blocker Cardioprotection

**Figure 1:**
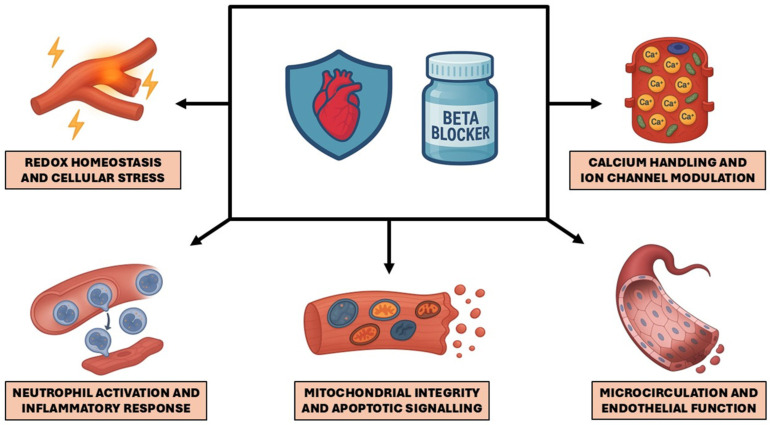
Summary of Beta-Blocker–mediated Cardioprotective Mechanisms.

Emerging experimental and translational data reclassify BBs as modulators of converging injury pathways rather than mere hemodynamic antagonists of catecholamine drive [46,47,48]. Most mechanistic signals derive from preclinical preparations: in vitro (cellular systems), ex vivo (perfused hearts), and in vivo (animal I/R models), while human evidence remains limited and largely indirect (biomarkers, intracoronary sampling, and imaging surrogates). Figure 1 provides a schematic overview of these mechanisms, and Table 1 maps the most studied BBs by generation to their reported effects based on current evidence.

Beta-blockers mitigate damage from ischemia and reperfusion by targeting several cellular and molecular processes: (1) maintenance of redox homeostasis and reduction in cellular stress; (2) attenuation of neutrophil activation and inflammatory responses; (3) preservation of mitochondrial integrity and regulation of apoptotic signaling; (4) improvement of microcirculatory dynamics and endothelial function; and (5) modulation of calcium handling and ion channel activity.

### 4.1. Redox Homeostasis and Cellular Stress

I/R imposes an abrupt, biphasic disruption of cellular redox homeostasis. During ischemia, electron transport is impaired and reductive pressure rises; with reperfusion, oxygen reintroduction accelerates electron leakage from mitochondrial complexes I and III and activates Nicotinamide Adenine Dinucleotide Phosphate (NADPH) oxidases (NOX), producing bursts of superoxide and hydrogen peroxide (H_2_O_2_) [46,49]. H_2_O_2_ can then participate in Fenton chemistry to generate hydroxyl radicals, initiating lipid peroxidation and protein/DNA injury; in parallel, reaction with Nitric Oxide (NO) yields peroxynitrite, which deranges signaling and impairs energy transfer [50,51]. These oxidant species perturb Ca^2+^ handling and Excitation–Contraction Coupling (ECC), promoting early contractile dysfunction [47,52,53]. Importantly, contemporary models argue that injury reflects not only excessive oxidation but also phases of reductive stress: oscillations in glutathione (GSH) and NAD(P)H redox couples alter the balance between adaptive redox signaling and overt damage [54]. This dynamic explains why untargeted antioxidant strategies have underperformed and suggests that effective cardioprotection depends on stabilizing, rather than abolishing, redox dynamics [54].

Against this backdrop, certain BBs exhibit agent-specific effects on redox control; carvedilol is the most thoroughly interrogated in this regard. Its carbazole moiety confers direct free-radical–scavenging activity and effectively inhibits lipid peroxidation by breaking chain reactions, complementing its β-adrenergic antagonism; these effects have been demonstrated in both cell-free and cellular systems [55]. In a canine low-flow ischemia and I/R model, pre-reperfusion carvedilol markedly reduced infarct size, and this protection was abolished by adenosine-receptor blockade or ecto-5′-nucleotidase inhibition, linking its benefit to relief of oxidative suppression of adenosine generation and restoration of an endogenous anti-ischemic signal at reperfusion [56]. These in vivo findings align with carvedilol’s capacity to temper oxidative bursts at their most injurious phase, rather than extinguishing physiological ROS signaling altogether. At the receptor level, carvedilol has been shown to uniquely prevent the so-called “redox inactivation” phenomenon: the selective downregulation of β1-adrenergic receptors by ROS, a property not shared by atenolol, metoprolol, timolol, or propranolol in side-by-side comparisons in isolated cardiomyocyte cultures [57].

Although less extensively characterized, redox-modulatory effects have also been reported for other agents. In a preclinical I/R model, chronic propranolol administration to murine pre-reperfusion improved post-ischemic recovery, reduced myocardial lipid peroxidation, and increased basal activities of catalase and GSH Peroxidase (GPx), without changes in Superoxide Dismutase (SOD) activity or antioxidant enzyme mRNA expression [58]. Notably, these protective effects persisted despite the absence of residual β-blockade, suggesting an induced antioxidant milieu rather than acute receptor antagonism, consistent with a regimen- and timing-dependent “antioxidant pre-conditioning” effect [58]. Moreover, bisoprolol’s redox effects have been best characterized in chronic post-ischemic/remodeling contexts but map onto processes that are highly pertinent to reperfusion injury. In the TO-2 hamster model of dilated cardiomyopathy, bisoprolol normalized myocardial redox indices, stabilized myocardial redox tone (normalizing the GSH/GSSG ratio), curtailed lipid peroxidation and protein nitration (4-HNE, 3-nitrotyrosine), and improved recovery of mitochondrial Manganese SOD (MnSOD) abundance [59]. These benefits occurred without suppression of NADPH oxidases (NOX) activity, implicating reinforcement of mitochondrial antioxidant defenses. Concurrent reductions in IL-1β and TNF-α further underscore the redox–inflammation crosstalk central to adverse post-I/R remodeling [59].

Finally, it is important to note that biased β-adrenergic signaling can, under certain conditions, shift toward maladaptive outcomes. For instance, metoprolol, despite its anti-inflammatory and Ca^2+^-regulatory properties (described below), has been shown to engage a GRK5/β-arrestin-2–dependent pathway in specific settings, promoting fibrotic gene expression and impairing diastolic function in vivo [60]. This highlights that redox-adjacent signaling bias can be either protective or detrimental, reinforcing the importance of both agent selection and timing in relation to reperfusion.

### 4.2. Neutrophil Activation and Inflammatory Response

I/R provokes a sterile inflammatory response in which neutrophils are early and decisive effectors. Reperfusion upregulates endothelial adhesion molecules and fosters platelet–leukocyte (especially platelet–neutrophil) aggregates, promoting intravascular stasis and capillary plugging that impede tissue perfusion [61,62]. Degradation of the endothelial glycocalyx, facilitated by TNF-α, further eases leukocyte and platelet adherence and amplifies inflammatory edema [63,64]. These events link neutrophil recruitment and activation to downstream microcirculatory dysfunction and expansion of injury.

Against this background, early pre-reperfusion β-blockade has been reappraised as an anti-inflammatory cardioprotective strategy. Translation has been inconsistent, with timing of therapy and ancillary properties repeatedly cited as key modifiers [48,65]. Building on this, it has been proposed that metoprolol attenuates neutrophil function and capillary plugging, thereby limiting Microvascular Obstruction (MVO) and final infarct size, although the current evidence base is weakened by the lack of direct MVO quantification and head-to-head comparisons with third-generation BBs [48]. Consistent with this view, direct experimental evidence has corroborated a non-class, neutrophil-targeted action of metoprolol [66]. In a 45 min/24 h murine I/R model, only metoprolol (not atenolol or propranolol) reduced infarct size, lowered myocardial neutrophil density, and decreased neutrophil–platelet interactions. Metoprolol uniquely inhibited neutrophil migration in vitro (CXCL1-induced transwell) and in vivo and reduced Neutrophil Extracellular Trap (NET) formation, as evaluated by dedicated indices (histone-3 citrullination, neutrophil elastase, myeloperoxidase) [66]. Two- and three-dimensional intravital microscopy demonstrated disrupted neutrophil crawling dynamics and impaired polarization exclusively with metoprolol, while complementary in silico analyses suggested that metoprolol–β1-adrenergic receptor binding enlarges the intracellular receptor cavity and exposes G Protein-Coupled Receptor (GPCR) Related Kinase (GRK) phosphorylation sites, a configuration potentially favoring β-arrestin–linked signaling consistent with a neutrophil-“stunning” phenotype [67].

Beyond leukocytes, metoprolol has been further shown to exert a direct anti-inflammatory effect on human-induced Pluripotent Stem Cell (iPSC)-derived cardiomyocytes exposed to TNF-α [downregulating Nuclear Factor-kappa B (NF-κB), IL-1β, IL-6, and Vascular Cell Adhesion Molecule-1 (VCAM-1)]. The effect was absent with esmolol and was abrogated by β-arrestin-2 knockdown, again consistent with β-arrestin-biased signaling at β1-receptors [68].

Interestingly, further in vitro work supports adrenoceptor-independent neutrophil modulation by BBs: metoprolol dose-dependently inhibited formyl-Methionyl-Leucyl-Phenylalanine (fMLP)-stimulated chemotaxis and formyl-peptide/phorbol myristate acetate–triggered superoxide release (whereas atenolol was inactive) [69]. In parallel, work with other BBs used topically showed that metipranolol and timolol inhibit neutrophil chemotaxis at micromolar concentrations, suppress fMLP-triggered respiratory burst at higher concentrations, and antagonize Protein Kinase C (PKC)–dependent radical generation [70]. Consequently, adrenoceptor-independent restraint of neutrophil migration and oxidant release by BBs warrants further investigation across a broader range of agents.

### 4.3. Mitochondrial Integrity and Apoptotic Signaling

Apoptosis is a prominent, early component of I/R Injury (IRI), particularly within border-zone tissue that is viable yet jeopardized. During reperfusion, ROS surges and Ca^2+^ overload converge at the mitochondria to trigger opening of the mitochondrial Permeability Transition Pore (mPTP) and activate stress-kinase programs [c-Jun N-terminal Kinase/Stress-Activated Protein Kinase (JNK/SAPK), p38 Mitogen-Activated Protein Kinase (p38 MAPK), NF-κB], culminating in caspase-mediated myocyte death [71,72,73]. This apoptotic burden accelerates contractile decline and sets the stage for adverse remodeling.

Among BBs, carvedilol has demonstrated the most consistent anti-apoptotic effects in preclinical models. In a lapine I/R preparation, a single i.v. dose given 5 min before reperfusion markedly reduced cardiomyocyte apoptosis (DNA fragmentation and nucleosomal laddering), attenuated SAPK activation by 50%, and blunted Fas upregulation; the effects were not replicated by propranolol [74]. An added aspect of protection comes from post-transcriptional regulation; in vivo, carvedilol restored cardiac miR-133 [a muscle-specific microRNA (myomiR) essential for normal function expression in oxidant-stressed cardiomyocytes, while repressing caspase-9/-3 [75]. A parallel miR axis has also been described: carvedilol downregulated pro-apoptotic miR-1 while upregulating the mitochondrial chaperone HSP60, tilting the Bcl-2/Bax balance toward survival and further constraining caspase-dependent death [76]. Complementing the acute setting, chronic pretreatment in a murine post-MI model, later subjected ex vivo to cardioplegia-type hypoxia/reperfusion, reduced peri-infarct apoptosis and preserved contractility through Phosphoinositide 3-Kinase (PI3K)- and MAPK Kinase (MEK)-dependent (but not PKA-mediated) signaling. This was accompanied by lower TNF-α/IL-8 expression and a shift in mitochondrial death mediators, with increased Bcl-2 and decreased cytochrome c [77]. Collectively, such findings reinforce mitochondria as a central therapeutic target for BB-mediated cardioprotection in the IRI setting.

### 4.4. Microcirculation and Endothelial Function

IRI involves not only cardiomyocyte death but also a spectrum of coronary microvascular damage; endothelial dysfunction with impaired vasomotion, increased vascular permeability, microembolization of atherothrombotic debris, and, in its most severe form, capillary destruction with hemorrhage [78]. These alterations culminate in Microvascular Obstruction (MVO) and no-reflow, which carry adverse prognostic significance [79,80,81]. Superoxide generated during reperfusion reacts rapidly with endothelial NO to form peroxynitrite, depleting NO bioavailability and impairing vasomotion; sustained oxidant load further “uncouples” endothelial NO synthase (eNOS) through oxidation of its tetrahydrobiopterin (BH4) cofactor, amplifying ROS production from the enzyme itself and perpetuating the vasoconstriction–edema vicious cycle [82].

Clinically oriented syntheses concur that among BBs, improvements in endothelial function are largely confined to third-generation agents with ancillary vasodilator/antioxidant properties, with nebivolol repeatedly demonstrating NO-dependent vasodilation and evidence of NADPH Oxidase (NOX) restraint across experimental work and human physiology studies [83]. Nebivolol is distinguished by endothelium-directed actions that favor NO bioavailability: beyond β1-selective antagonism, it engages endothelial pathways to enhance eNOS activity and NO/cGMP signaling and, in parallel, lowers Endothelin-1 (ET-1) via reduction in preproendothelin-1 mRNA levels [84,85]. Furthermore, nebivolol has been found to reduce proliferation of coronary endothelial and smooth-muscle cells, an effect not reproduced by propranolol, metoprolol, or bisoprolol under the same conditions [85,86]. At the transcriptional level, nebivolol downregulates inflammatory and pro-proliferative programs in human coronary artery smooth-muscle cells [including IL-1α, Cyclooxygenase (COX)-2, Growth-Related Oncogene (GRO) β/γ, Platelet-Derived Growth Factor (PDGF-A), and Monocyte Chemoattractant Protein (MCP)-1] concomitant with reduced NF-κB activity and lower MCP-1 release, with metoprolol showing the opposite direction for several of these genes [87].

Carvedilol, while best known for direct ROS scavenging and adenosine-linked reperfusion benefit (Section 4.1), also acts favorably at the endothelium: it improves flow-mediated dilation in hypertensive patients (including those with diabetes) and outperforms metoprolol on endothelial endpoints in head-to-head physiological studies, consistent with an antioxidant mechanism that preserves NO signaling during and after ischemia/reperfusion [83]. Complementing these endothelial effects, carvedilol, like nebivolol, also suppresses ET-1 output in human coronary cells, aligning with a net shift toward vasodilator predominance [85].

By contrast, first- and second-generation β1-selective agents without endothelial agonism generally appear neutral on NO-dependent vasodilation and arterial structure in studies; atenolol and metoprolol have not consistently improved endothelial function [88,89,90,91].

### 4.5. Calcium Handling and Ion Channel Modulation

ECC depends on a tightly regulated sequence in which depolarization opens L-type Ca^2+^ Channels (LTCC/Cav1.2) within T-tubules to admit trigger Ca^2+^, evoking Ca^2+^-induced Ca^2+^ release via Ryanodine Receptor-2 (RyR2) on the sarcoplasmic reticulum (SR); relaxation then requires Ca^2+^ reuptake by SERCA2a and extrusion by the Na^+^/Ca^2+^ exchanger (NCX) [92,93,94,95]. During ischemia, intracellular acidosis stimulates Na^+^/H^+^ exchange (HNX), increasing Na^+^ ions and driving reverse-mode NCX Ca^2+^ influx; upon reperfusion and extracellular alkalinization, both HNX and NCX further amplify cytosolic Ca^2+^ [96]. ROS damage sarcolemmal and SR membranes and destabilize RyR2, exacerbating diastolic SR Ca^2+^ leak [97,98]. At the organ level, Ca^2+^ overload contributes to arrhythmias, contractile failure, and microvascular injury with inflammatory cell recruitment [99].

Within this framework, β_1_-adrenergic blockade targets the upstream trigger of Ca^2+^ overload. By dampening Gs-cAMP signaling, it reduces Protein Kinase A (PKA)—and Ca^2+^/Calmodulin-dependent protein Kinase II (CaMKII)–dependent phosphorylation at the principal ECC nodes (LTCC, RyR2, PLB) and improves SERCA2a-mediated reuptake [100]. In practical terms, β-blockade attenuates reperfusion-phase Ca^2+^ overload by simultaneously reducing Ca^2+^ influx, SR leak, and defective reuptake across this network. Consistent with this mechanism, metoprolol is explicitly listed among six agents positioned at the intersection of myocardial IRI and the calcium signaling pathway (alongside adenosine, ridogrel, vorapaxar, flunarizine, and zoniporide), with the β_1_-adrenergic receptor enumerated among relevant targets together with voltage-dependent Ca^2+^ channels, Na^+^/H^+^ exchange (HNX), PAR1, adenosine A2B receptor, and thromboxane A_2_ receptors [101].

## 5. Current Beta-Blocker Positioning in Guidelines and Data from Clinical Studies

### 5.1. Current Position in Guidelines

Current American Heart Association guidelines recommend early initiation of BBs after ST-Elevation MI (Class I, Level of Evidence A) to reduce the risk of reinfarction and ventricular arrhythmias, unless contraindicated [102]. Intravenous BBs may also be considered in selected patients, where benefit has been demonstrated [103]. The European Society of Cardiology similarly provides a Class I recommendation (Level A) for BB use after ACS in patients with LVEF ≤ 40%, independent of HF symptoms [104]. However, both sets of guidelines acknowledge persistent uncertainty regarding the role of BBs in Non-ST-Elevation MI and the optimal duration of therapy in patients with preserved EF, as recent trials in the modern reperfusion era have not provided conclusive answers [105]. By contrast, the benefits of BBs in patients with reduced EF after ACS are firmly established in landmark trials, including CIBIS-II (bisoprolol), MERIT-HF (metoprolol succinate), and COPERNICUS (carvedilol), all of which demonstrated improvements in survival, morbidity, and reverse remodeling in patients with systolic dysfunction [106,107,108].

### 5.2. Recent Clinical Data

Recent large-scale trials now challenge the long-standing assumption that all post-MI patients benefit from BBs, particularly in the modern era of routine reperfusion, new-generation stents, advanced revascularization techniques, and optimized medical therapy. The REBOOT-CNIC trial (>8400 patients; median follow-up 3.7 years; EF > 40%) found no reduction in death, reinfarction, HF hospitalization, or adverse safety events with BBs compared to placebo [109]. Similarly, the BETAMI-DANBLOCK trial (5574 patients; median follow-up 3.5 years; EF > 40%) showed only a modest reduction in composite endpoints (14.2% vs. 16.3%), driven mainly by fewer reinfarctions, with no difference in mortality or other outcomes. Importantly, both trials suggest that any potential benefit may be limited to patients with mildly reduced EF (40–49%), while those with preserved EF appear to gain little or no advantage.

Likewise, the REDUCE-AMI trial (>5000 patients, mostly from Sweden, with EF ≥ 50%) demonstrated no difference in the primary composite endpoint (7.9% vs. 8.3%; HR 0.96, *p* = 0.64) or in safety outcomes between carvedilol and placebo [110]. These findings are consistent with smaller studies. A Japanese randomized control trial of ~800 acute MI patients with EF > 40% and a median follow-up of 3.9 years showed no benefit of carvedilol compared with placebo [111]. Lastly, in the CHARISMA trial, BBs lowered recurrent MI risk in prior MI patients without HF (3.4% vs. 4.9%; HR 0.62) but did not improve survival (5.3% vs. 6.7%). In patients without MI, no benefit was seen, and stroke risk was higher (3.5% vs. 1.5%; HR 2.69) [112].

### 5.3. Imaging and Functional Data

Beyond their proven survival and mortality benefits, BBs have also shown favorable effects on cardiac structure and function as assessed by imaging modalities [113]. These trials are particularly valuable when they include patients across the spectrum of systolic function [114]. The subgroup with HF with preserved EF (HFpEF) remains of special interest, as evidence is limited and significant knowledge gaps persist, making studies in this population especially important. It is also important to note that the efficacy of BBs has partly been attributed to their ability to lower heart rate, which reduces myocardial oxygen demand, prolongs diastolic filling time, and improves coronary perfusion [115]. This bradycardic effect contributes to their protective role in ischemia and remodeling, although its benefit may vary depending on baseline ventricular function [116].

#### 5.3.1. Imaging Outcomes in Systolic Dysfunction

In the REVERT trial, 149 asymptomatic patients with LV systolic dysfunction (EF < 40%) were randomized to metoprolol succinate (200 mg or 50 mg) or placebo for 12 months. High-dose therapy (200 mg) significantly improved cardiac remodeling, reducing LV end-systolic volume index by ~14 mL/m^2^ and increasing LVEF by ~6%, while the lower dose showed more modest effects [117]. In a double-blind, placebo-controlled trial from the CHAPS database, 49 post-MI patients with LVEF < 45% were randomized to carvedilol or placebo for 6 months. Echocardiographic assessment showed that carvedilol significantly attenuated adverse remodeling: it reduced wall thickness opposite the infarct (−1.3 mm vs. +0.6 mm, *p* = 0.01), decreased LV mass (−18 g vs. +25 g, *p* = 0.02), preserved sphericity index, and prevented progression of wall thickening abnormalities at the infarct site [118]. In the CAPRICORN Echocardiographic substudy, carvedilol added to ACE inhibitors improved LV end-systolic volume and LVEF in patients with ventricular dysfunction after MI [119]. Carvedilol has also shown similar results in cardiac magnetic resonance study in stable patients with chronic HF. BBs have shown benefit in parameters of the right ventricle in the same group of patients [120].

#### 5.3.2. Imaging Outcomes in Preserved Systolic Function

In a large Japanese registry, 602 matched HFmrEF (LVEF 40–49%) patients with dilated cardiomyopathy were analyzed for reverse remodeling. After 2 years, ≥10% LVEF increase was more frequent with BBs (24.3% vs. 17.8%; OR 1.48, *p* = 0.006). Benefits were strongest in patients with HR ≥ 75 bpm and those with atrial fibrillation [121]. In a trial with 114 patients with EF ≥ 40% with complete revascularization after AMI, BBs did not show significant changes in LVEF, left ventricular end-diastolic volume index or left ventricular end-systolic volume index [122].

### 5.4. Markers of Myocardial Injury

In a study with CAD patients with HF, adding sacubitril/valsartan to metoprolol improved endothelial function beyond metoprolol alone, as shown by lower Endothelin-1 and higher NO levels. These changes, alongside reduced cardiac troponin I and N-terminal pro–B-type natriuretic peptide, indicate that combined therapy not only limits cardiac stress and remodeling but also enhances vascular protection [123].

### 5.5. Other Results

BBs are also recommended for the treatment of stable angina uncomplicated by bradycardia, tachycardia, left ventricular dysfunction, or hypotension [124,125]. They also improve diagnostic imaging, as precise heart-rate control enhances image quality during computed tomography coronary angiography while maintaining hemodynamic safety [115].

## 6. Limitations and Future Research Directions

Despite extensive preclinical and clinical research, important limitations persist in the literature regarding BBs and cardioprotection against adverse remodeling. Many mechanistic insights have not translated into consistent clinical benefit, reflecting both challenges in study design and the inherent complexity of myocardial remodeling [126]. Conventional animal I/R models often fail to reflect current clinical practice, as they typically use young, otherwise healthy animals and prolonged ischemic times [127]. In contrast, contemporary patients usually undergo rapid reperfusion and receive multiple cardioprotective therapies, including potent antiplatelet agents and high-intensity statins, which may diminish the relative impact of beta-blockers observed in preclinical studies [128].

A key unresolved issue is not only the optimal timing for BBs initiation but also the potential need for discontinuation, since efficacy may vary across ischemia, reperfusion, and chronic remodeling phases, each driven by distinct pathophysiologic mechanisms [129]. Early BB use appears beneficial by reducing reperfusion injury, neutrophil infiltration, and oxidative stress, while later use mainly affects remodeling rather than infarct size [117,118,119]. Evidence is still inconsistent in later administration or broader patient groups. Although, recent studies suggest that continuation beyond one year in patients without LV dysfunction may have limited benefit [109,110,111]. Consequently, the optimal timing remains unresolved and highlights the need for further trials.

Future trials must also clarify whether all BBs confer comparable protection, as conformational differences in receptor binding suggest drug-specific effects. Another major limitation is the lack of standardized characterization of their cardioprotective actions—whether predominantly antioxidant, anti-inflammatory, or anti-fibrotic—making cross-study comparisons difficult [130]. Although BBs exhibit pleiotropic properties, including antioxidant and anti-inflammatory effects, there remains ongoing debate regarding whether the timing, strength, and clinical relevance of these actions are sufficient to impact hard endpoints like mortality or recurrent infarction when added to optimized, contemporary therapies. Definitive answers to this question will depend on rigorously designed randomized controlled trials targeting well-defined patient cohorts and comprehensive mechanistic studies. When broader patient groups—especially those lacking left ventricular dysfunction—are included in studies, the results have so far failed to demonstrate meaningful improvements in major outcomes [131].

The pronounced hemodynamic actions of BBs—such as lowering HR and BP—can play a meaningful role in driving the observed clinical benefits, yet they may also overshadow more subtle molecular effects during clinical trials. This limitation is particularly evident in studies where hard outcomes are heavily influenced by procedural success and comprehensive background pharmacotherapies, making it difficult to distinguish the incremental advantages conferred by BBs at the cellular or molecular level [132].

Addressing these gaps will be critical for refining the role of BBs in adverse remodeling and for guiding their use in specific patient subgroups, including those with preserved ventricular function [133].

## 7. Conclusions

BBs provide cardioprotection beyond their primary hemodynamic effects, in an agent-specific manner. Evidence implicates mitigation of cellular redox stress and inflammation, preservation of mitochondrial function, support of anti-apoptotic signaling, improved endothelial nitric oxide bioavailability, and stabilization of calcium handling. Third-generation agents (carvedilol, nebivolol) most consistently demonstrate antioxidant and endothelial benefits.

In practice, BBs remain standard after MI when reduced EF is present, whereas contemporary trials in reperfused patients with preserved EF show neutral or modest benefit. These data favor a targeted strategy to match the agent to the clinical substrate (e.g., EF, resting heart rate, arrhythmic risk, microvascular dysfunction) and tailor timing, dose, and route—including pre-, peri- and post-percutaneous coronary intervention use when appropriate.

Future work should prioritize standardized methods in preclinical studies; head-to-head clinical trials with harmonized mechanistic and imaging endpoints (e.g., microvascular obstruction and infarct characteristics on cardiac magnetic resonance); clearer criteria for continuation versus withdrawal in the chronic phase; and rational combinations with therapies acting on complementary pathways.

## Figures and Tables

**Table 1:** Summary of BB effects across key mechanistic pathways of IRI based on preclinical and translational evidence.

Gen	Agent	Redox Homeostasis	Neutrophil/ Inflammation	Mitochondria and Apoptosis	Microcirculation/ Endothelium	Ca^2+^ Handling/ECC
**F** **I** **R** **S** **T**	**Propranolol**	+ (chronic pre-conditioning: ↓lipid peroxidation; catalase/GPx ↑; β-blockade-independent)	−/± (limited; context-specific)	− (no clear signal)	− (no clear signal)	+ (class β1 effect)
**S** **E** **C** **O** **N** **D**	**Atenolol**	− (no clear signal)	− (no neutrophil effects in comparisons)	− (no clear signal)	− (neutral on endothelial function)	+ (class β1 effect)
	**Bisoprolol**	+ (GSH/GSSG normalization; 4-HNE/3-NT ↓; MnSOD recovery)	− (no clear signal)	+ (Bcl-2↑; cytochrome c↓)	− (no clear signal)	+ (class β1 effect)
	**Metoprolol**	−/± (context-dependent bias)	++ (β1/β-arrestin–biased neutrophil “stunning”; ↓NETs/adhesion/migration)	− (no consistent evidence)	− (no consistent endothelial gain)	+ (attenuates reperfusion Ca^2+^ overload; listed among Ca^2+^-pathway agents)
**T** **H** **I** **R** **D**	**Carvedilol**	++ (direct scavenging; prevents β1 “redox inactivation”; adenosine restoration)	− (no clear signal)	++ (↓SAPK/Fas; miR-133↑/miR-1↓; ↓caspase-9/-3; Bcl-2/Bax→survival)	+/++ (↑NO bioavailability; ↓ET-1; flow-mediated dilation ↑)	+ (β1 block; stabilizes SR leak indirectly)
	**Nebivolol**	+ (NOX restraint, indirect)	− (no clear signal)	− (no clear signal)	++ (eNOS/NO–cGMP ↑; ET-1 ↓; anti-proliferative)	+ (class β1 effect)

Symbols denote the strength and nature of evidence: ++ strong supportive evidence; + supportive/indirect evidence; − neutral or insufficient evidence; ± mixed or timing/setting-dependent evidence; −/± largely neutral with limited, context-specific signals. Symbols: ↑: increase; ↓: decrease. Parenthetical text describes principal mechanistic features or study findings underpinning each entry. 3-NT: 3-Nitrotyrosine, 4-HNE: 4-Hydroxy-2-Nonenal, BB: Beta Blocker, ECC: Excitation–Contraction Coupling, eNOS: endothelial Nitric Oxide Synthase, ET-1: Endothelin-1, Gen: Generation, GPx: Glutathione Peroxidase, GSH: Reduced Glutathione, GSSG: Oxidized Glutathione, IRI: Ischemia/Reperfusion Injury, miR: microRNA, MnSOD: Manganese Superoxide Dismutase, NETs: Neutrophil Extracellular Traps, NO: Nitric Oxide, NOX: NADPH Oxidase, SAPK: Stress-Activated Protein Kinase, SR: Sarcoplasmic Reticulum.

## Data Availability

No new data were created or analyzed in this study. Data sharing is not applicable to this article.

## Abbreviations

The following abbreviations are used in this manuscript:

4-HNE	4-hydroxynonenal	IHD	ischemic heart disease
AEBP1	adipocyte enhancer -binding protein 1	IL	interleukin
ACS	acute coronary syndrome	iPSC	induced pluripotent stem cell
AMI	acute myocardial infarction	IRI	ischemia/reperfusion injury
AP	angina pectoris	JNK	c-Jun N-terminal kinase
AT1	angiotensin II type 1 receptor	LTCC	L-type calcium channel
AT2	angiotensin II type 2 receptor	LV	left ventricle
ATP	adenosine triphosphate	LVEF	left ventricular ejection fraction
BB	beta-blocker	MACE	major adverse cardiovascular events
BH4	tetrahydrobiopterin	MAPK	mitogen-activated protein kinase
BP	blood pressure	MCP-1	monocyte chemoattractant protein-1
CAD	coronary artery disease	MEK	MAPK kinase
CaMKII	Calcium/calmodulin- dependent protein kinase II	miR	microRNA
cAMP	cyclic adenosine monophosphate	miRNA	microRNA
cGMP	cyclic guanosine monophosphate	MnSOD	manganese superoxide dismutase
CILP1	cartilage intermediate layer protein 1	mPTP	mitochondrial permeability transition pore
CNS	central nervous system	MVO	microvascular obstruction
COX-2	cyclooxygenase-2	NADPH	nicotinamide adenine dinucleotide phosphate
CXCL1	C-X-C motif chemokine ligand 1	NCX	sodium/calcium exchanger
DNA	deoxyribonucleic acid	NET	neutrophil extracellular trap
ECC	excitation–contraction coupling	NF-κB	nuclear factor kappa-light-chain- enhancer of activated B cells
ECM	extracellular matrix	NO	nitric oxide
EF	ejection fraction	NOX	NADPH oxidase
eNOS	endothelial nitric oxide synthase	PAR1	protease-activated receptor 1
ET-1	endothelin-1	PDGF-A	platelet-derived growth factor A
fMLP	formyl-Methionyl-Leucyl- Phenylalanine	PI3K	phosphoinositide 3-kinase
Gi	inhibitory G protein	PKA	protein kinase A
GPCR	G protein-coupled receptor	PKB	protein kinase B
GPx	glutathione peroxidase	PKC	protein kinase C
GRK	G protein-coupled receptor kinase	PLB	phospholamban
GRO	growth-related oncogene	RAAS	renin–angiotensin–aldosterone system
Gs	stimulatory G protein	ROS	reactive oxygen species
GSH	reduced glutathione	RyR2	ryanodine receptor 2
GSSG	oxidized glutathione	SAPK	stress-activated protein kinase
H_2_O_2_	hydrogen peroxide	SERCA2a	sarcoplasmic/endoplasmic reticulum Ca^2+^-ATPase 2a
HF	heart failure	SOD	superoxide dismutase
HFmrEF	heart failure with mildly reduced ejection fraction	SNS	sympathetic nervous system
HFpEF	heart failure with preserved ejection fraction	SR	sarcoplasmic reticulum
HFrEF	heart failure with reduced ejection fraction	TGFBI	transforming growth factor β-induced
HNX	Na^+^/H^+^ exchanger	TLR	toll-like receptor
HR	heart rate	TNF-α	tumor necrosis factor alpha
HSP60	heat shock protein 60	VCAM-1	vascular cell adhesion molecule-1
I/R	ischemia/reperfusion		
